# Accuracy of US College Football Players’ Estimates of Their Risk of Concussion or Injury

**DOI:** 10.1001/jamanetworkopen.2020.31509

**Published:** 2020-12-29

**Authors:** Christine M. Baugh, Emily Kroshus, William P. Meehan, Thomas G. McGuire, Laura A. Hatfield

**Affiliations:** 1 Department of Health Care Policy, Harvard Medical School, Boston, Massachusetts; 2 Center for Bioethics and Humanities, University of Colorado Denver Anschutz Medical Campus, Aurora; 3 Division of General Internal Medicine, Department of Medicine, University of Colorado School of Medicine, Aurora; 4 Department of Pediatrics, University of Washington, Seattle; 5 Center for Child Health, Behavior and Development, Seattle Children’s Research Institute, Seattle, Washington; 6 Sports Concussion Clinic, Department of Sports Medicine, Boston Children’s Hospital, Boston, Massachusetts

## Abstract

**Importance:**

Despite increased concern about the health consequences of contact sports, little is known about athletes’ understanding of their own risk of sports-related injury.

**Objective:**

To assess whether college football players accurately estimate their risk of concussion and nonconcussion injury and to identify characteristics of athletes who misestimate their injury risk.

**Design, Setting, and Participants:**

In this survey study, questionnaires were given to 296 current college football players on 4 teams from the 3 of the 5 most competitive conferences of the US National Collegiate Athletic Association. Surveys were conducted between February and May 2017. Data were analyzed from June 2017 through July 2020.

**Main Outcomes and Measures:**

Multiple approaches were taken to compare athlete perceptions of their risks of concussion and nonconcussion injury with individual probabilities of these risks, which were modeled using logistic regression.

**Results:**

Of 296 male college-aged athletes from 4 football teams who participated in the survey, 265 (89%) answered all questions relevant for this study. Participating teams were similar to nonparticipating teams across nearly all measured characteristics. One hundred athletes (34%) had sustained 1 or more concussions, and 197 (68% of the 289 who responded to the question) had sustained 1 or more injuries in the previous football season. Logistic regression models of single-season injury and concussion had reasonably good fit (area under the curve, 0.75 and 0.73, respectively). Of the 265 participants for whom all relevant data were available, 111 (42%) underestimated their risk of concussion (χ^2^ = 98.6; *P* = .003). A similar proportion of athletes (113 [43%]) underestimated their risk of injury, although this was not statistically significant (χ^2^ = 34.0; *P* = .09). An alternative analytic strategy suggested that 241 athletes (91%) underestimated their risk of injury (Wilcoxon statistic, 7865; *P* < .001) and 167 (63%) underestimated their risk of concussion (Wilcoxon statistic, 26 768; *P* < .001).

**Conclusions and Relevance:**

The findings of this survey study suggest that college football players may underestimate their risk of injury and concussion. The implications for informed participation in sport are unclear given that people generally underestimate health risks. It is necessary to consider whether athletes are sufficiently informed and how much risk is acceptable for an athlete to participate in a sport.

## Key Points

**Question:**

Do US college football players accurately estimate their risk of injury or concussion?

**Findings:**

In this survey study of 296 male college-aged athletes, estimates from analytic strategies comparing modeled risks with athlete perceptions suggested that 43% of athletes underestimated their risk of injury and 42% underestimated their risk of concussion. Alternative analytic strategies suggested that 91% of athletes underestimated their risk of injury and 63% underestimated their risk of concussion.

**Meaning:**

In this study, many college football players underestimated their personal risk of concussion and other injury, raising ethical concerns about informed participation.

## Teaser

This survey study uses questionnaires collected from college football players in the most competitive conferences of the US National Collegiate Athletic Association to analyze whether the players accurately estimate their risk of concussion and nonconcussion injury.

## Introduction

Concern about the health consequences associated with repetitive brain trauma has led to debate regarding the acceptability of activities such as full-contact US football. One common perspective is that individuals should be allowed to make informed choices about their participation in such activities.^1,2^ One reason to value individual decision-making is that individuals are best positioned to evaluate risks and benefits in the context of their particular set of values and preferences. However, individuals making decisions in the absence of full information may not be able to act in accordance with their values and interests.^3^ That is, otherwise free choices based on poorly estimated risks and benefits may not be value enhancing.^4^ Another viewpoint is that risk reduction is partly the responsibility of policy makers and should not be left entirely to individuals.^5^ A policy approach can be helpful in cases in which power dynamics, information imbalances, or social circumstances may lead to an unjust distribution of risks among a population or when public health concerns outweigh individual liberty considerations. The tension between individual choice and collective responsibility to prevent injury and disease is a central tension in creating health-related laws and policies.^6^

Sound decision-making requires accurate information about risks and benefits. However, football players may underestimate their risk of injury owing to a combination of known tendencies: men, the main participants in football, perceive lower levels of risk than women^7^; individuals in general estimate their personal risk as less than that of the typical person^8^; people who participate in an activity view the risks as smaller than do nonparticipants^9^; and athletes perceive lower risks than do nonathletes.^10^ Lower risk perception is associated with more risk-taking behavior^11^; thus, underestimating risks may influence athletes’ decisions to participate in sport. Furthermore, perceived risk and subsequent injury are inversely associated; that is, athletes with low perceived risk may be at higher risk of subsequent injury.^12^ Whereas risk attitudes, or an individual’s willingness to take on a given amount of risk, tend to be subjective and difficult to modify, risk perceptions, or an individual’s view on how much risk is inherent in a given activity, are broadly objective and have the potential to be corrected through effective knowledge translation efforts.^13^ In aggregate, these findings support the existence of an association between athletes’ perceptions of risks and their decisions to participate in sports, that underestimation of risks may be associated with more injuries, and that misestimation of risks may be effectively addressed by educational intervention.

As athletes are asked to return to sport amid the coronavirus disease 2019 (COVID-19) pandemic, understanding whether they accurately appraise risk is of increased importance. If they misestimate their personal risk of well-understood hazards associated with football participation, it is likely that they may also misestimate the less well-understood risks associated with COVID-19.

In 2017, we conducted a cross-sectional survey of college football players in highly competitive programs. We hypothesized that athletes would underestimate their personal risks of injury and concussion. First, we modeled their single-season risk of overall injury and concussion specifically using injury history and athletic and demographic characteristics. Then we compared the modeled risks with athletes’ perceptions of their injury and concussion risks to evaluate the accuracy of athletes’ estimations. We also evaluated the characteristics of athletes who underestimated their injury and concussion risks.

## Methods

For this survey study, we invited 65 college football teams in the top 5 most competitive and well-funded conferences in the National Collegiate Athletic Association (Power 5 Conferences): Atlantic Coast Conference, Big Ten Conference, Big 12 Conference, Pacific-12 Conference, and Southeastern Conference. Four teams agreed to allow their athletes to participate between February and May 2017. All athletes were provided with an informed consent form and information about the study and were given an opportunity to ask questions. After this process, consent was implied by completing the questionnaire. This process was approved by the Harvard T.H. Chan School of Public Health institutional review board. Athletes who chose to participate completed a 15-minute questionnaire and were provided a $10 Amazon gift card. This study followed the American Association for Public Opinion Research (AAPOR) reporting guideline.

### Measures

#### Athlete Risk Perceptions, Single-Season Injury, and Injury History

Athletes estimated the likelihood of sustaining a concussion and/or a nonconcussion injury during the following season using an ordinal scale ranging from “definitely won’t” (1) to “definitely will” (7). Injury was defined as harm to the body that requires medical attention.

A binary indicator for previous concussion was created from responses to the following question: “During the previous football season, how many times did you think you had a concussion?” The same was done for injury.

Athletes’ injury history before the previous football season was computed using responses to the following questions: “During your football career, how many times did you think you had a concussion?” “During your football career, how many times did you think you had an injury that was not a concussion?” Injury and concussion in the previous football season were subtracted.

#### Athlete Characteristics

Athletes indicated their primary playing position, years of full-contact football participation, role on the team (eg, first-team or starter, second-team or back-up), year on the team (eg, first-year athlete), race/ethnicity, and maternal and paternal educational attainment.

### Statistical Analysis

Data were analyzed from June 2017 through July 2020. First, we compared athletes’ perceived likelihood of future concussion and future injury separately with the rates of these outcomes in the previous season. We grouped athletes into 3 categories based on their responses on the 7-point Likert scale measuring perceptions: “likely” (5-7), “neither likely nor unlikely” (4), and “unlikely” (1-3).

Using logistic regressions, we modeled the single-season risk of concussion and injury separately with the following factors: year on team, primary playing position, role on team, years playing football, team, and injury history. These models produced estimated individual probabilities of concussion and injury.

We categorized the modeled probabilities into 7 bins for comparison with athlete perceptions on the ordinal scale. First, we used literature on how people interpret numerical probabilities (hereafter, *literature-derived cut points*).^14^ Second, we chose cut points that minimized the difference between model-estimated probabilities and athlete perceptions (hereafter, *data-driven cut points*). We also transformed athlete perceptions into numerical probabilities using similar procedures (full details given in the eAppendix in the Supplement).

We tested for independence between the categorized modeled probabilities and athlete perceptions using a χ^2^ test. We tested for a difference in the distribution of modeled probabilities and transformed athlete perceptions using a Wilcoxon signed rank test. Subtracting the modeled probabilities from the transformed athlete perceptions yielded a discrepancy measure; negative values indicated that athletes underestimated risk compared with the model. We summarized characteristics of athletes with overestimated and underestimated risks and fit linear regression models to risk discrepancies with athlete characteristics as factors.

We used publicly available information^15^ to compare participating and nonparticipating teams for possible school- or team-level differences using *t* tests (continuous variables) or χ^2^ tests (categorical variables). The significance level was set at *P* < .05 using a 2-sided test. Analyses were conducted using R statistical software, version 3.5.1 (R Project for Statistical Computing).

## Results

### Sample

Of the 296 college-aged male athletes who participated in this survey study, 150 (51%) were non-Hispanic White individuals; 265 athletes (89%) answered all questions relevant for this study. Within-team participation ranged from 64% to 100%, the overall response rate was 89%, and the effective response rate was 80%. Across many observable characteristics, participating teams did not differ significantly from nonparticipating teams (Table 1). However, there was heterogeneity between participating and nonparticipating teams in the mean (SD) football team size (participating teams, 114 [2.38] athletes; nonparticipating teams, 120 [8.97] athletes; *P* = .003).

**Table 1.** Comparisons of Participating and Nonparticipating Teams and Schools

Variable	Mean (SD)		*t* statistic	*P* value
	Participating schools or teams (n = 4)	Nonparticipating schools or teams (n = 61)		
Undergraduate students				
Male	10 284 (5065)	11 146 (4925)	0.33	.76
Female	9880 (4561)	11 380 (4649)	0.64	.57
Intercollegiate athletes				
Male	315 (96)	359 (83)	0.91	.43
Female	317 (113)	337 (89)	0.35	.75
Men’s sports teams				
Head coach annual salary, $	1 037 355 (416 892)	1 191 202 (349 347)	−0.72	.52
Assistant coach annual salary, $	244 223 (36 554)	245 716 (73 012)	0.07	.95
Athletic student aid, $	8 363 334 (2 354 124)	7 945 395 (2 324 924)	−0.34	.75
Football				
Team athletes	114 (2.38)	120 (8.97)	3.82	.003
Total expenses, $	25 434 327 (5 483 434)	32 352 694 (10 842 558)	2.25	.08
Total operating expenses, $	4 202 291 (1 342 859)	5 633 419 (2 625 668)	1.91	.12
Total revenue, $	52 438 888 (20 985 441)	60 309 992 (29 110 439)	0.71	.52
Total school athletics, $				
Revenue	123 305 230 (38 006 225)	117 770 913 (34 535 220)	−0.28	.79
Expenses	107 435 134 (21 413 990)	110 675 915 (29 944 065)	0.29	.79
Operating expenses	11 354 516 (3 218 960)	14 118 723 (4 269 212)	1.63	.18
Football team wins/losses in 2017	0.550 (0.377)	0.561 (0.194)	0.06	.96

### Descriptive Comparison of Previous-Year Injury Rates and Athlete-Perceived Likelihoods

Of the 294 athletes who responded to the question about previous-season concussions, 100 (34%) had sustained at least 1 suspected concussion in the previous football season; the total number of suspected concussions in the previous season was 171, with a mean (SD) of 43 (6.5) suspected concussions per participating team. However, of the 291 athletes who responded to the question asking them to estimate the likelihood of sustaining a concussion in the next season, 26 (9%) thought it was likely, 174 (60%) thought it was unlikely, and 91 (31%) thought it was neither likely nor unlikely. A total of 197 athletes (68% of the 289 who responded to the question) indicated that they had sustained 1 or more injuries in the previous football season; the total number of suspected injuries in the previous season was 483. A total of 57 athletes (20% of the 290 who responded to the question) thought it was likely that they would incur an injury in the next season; 140 (48%) thought it was unlikely; and 83 (29%) thought it was neither likely nor unlikely.

### Transforming Probabilities to Categories

**Figure 1.**
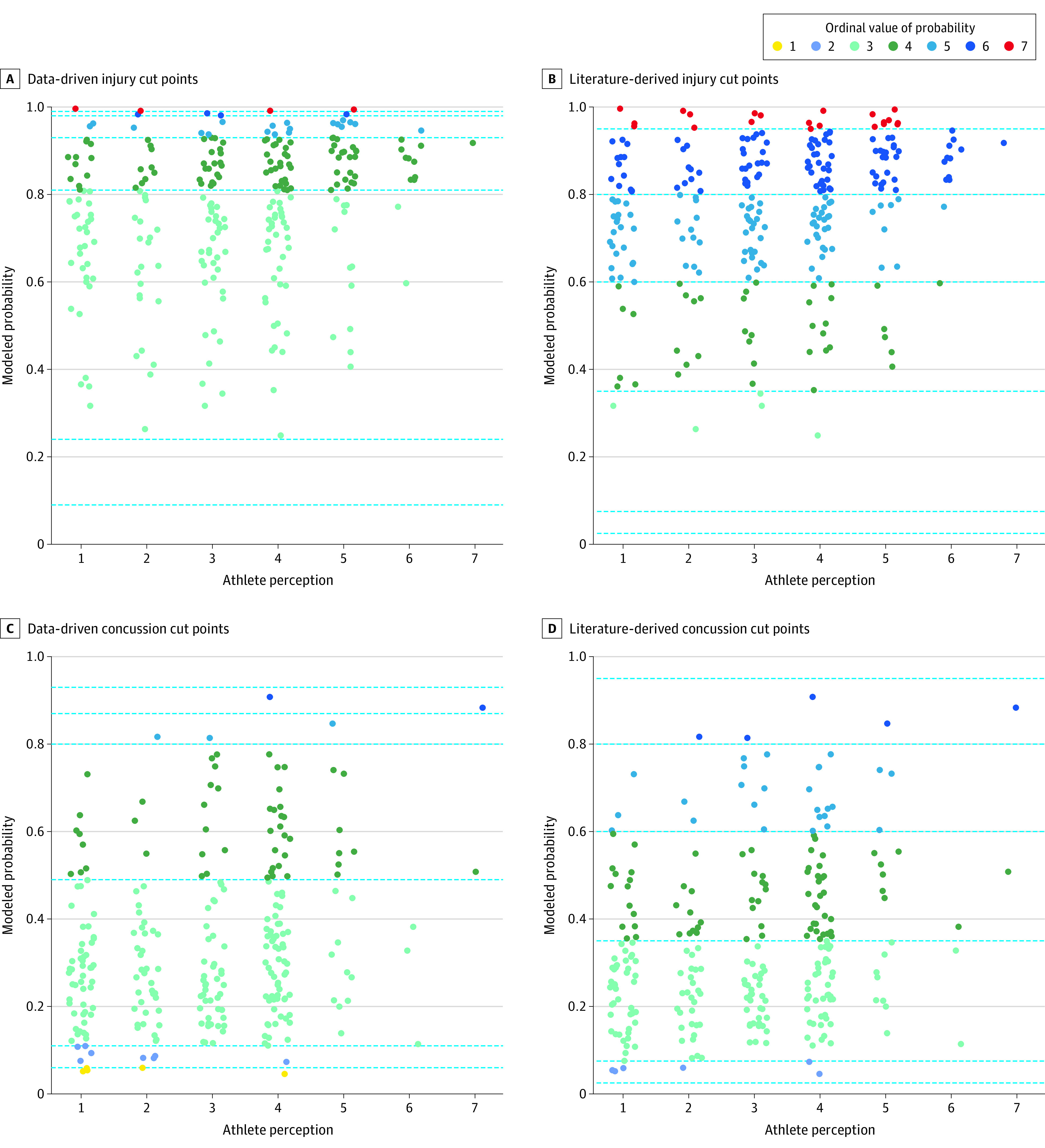
Athletes’ Perceived Risk of Injury or Concussion vs Modeled Single-Season Probabilities of Injury or Concussion A and C, The horizontal dashed lines indicate cut points based on minimizing discrepancy between athlete risk categories and categorized probabilities. B and D, The horizontal dashed lines indicate cut points from previous literature. The colors indicate the ordinal value assigned to each modeled probability using the cut points.

Literature-derived and data-driven approaches to transforming probabilities into categorical data produced different categorizations (Figure 1). The data-driven cut points created middle categories with wide ranges, thereby assigning many individuals to these categories (Figure 1A and C), whereas the literature-derived cut points assigned more modeled probabilities to the higher and lower categories (Figure 1B and D).

### Model-Based Injury Risk Estimation

**Figure 2.**
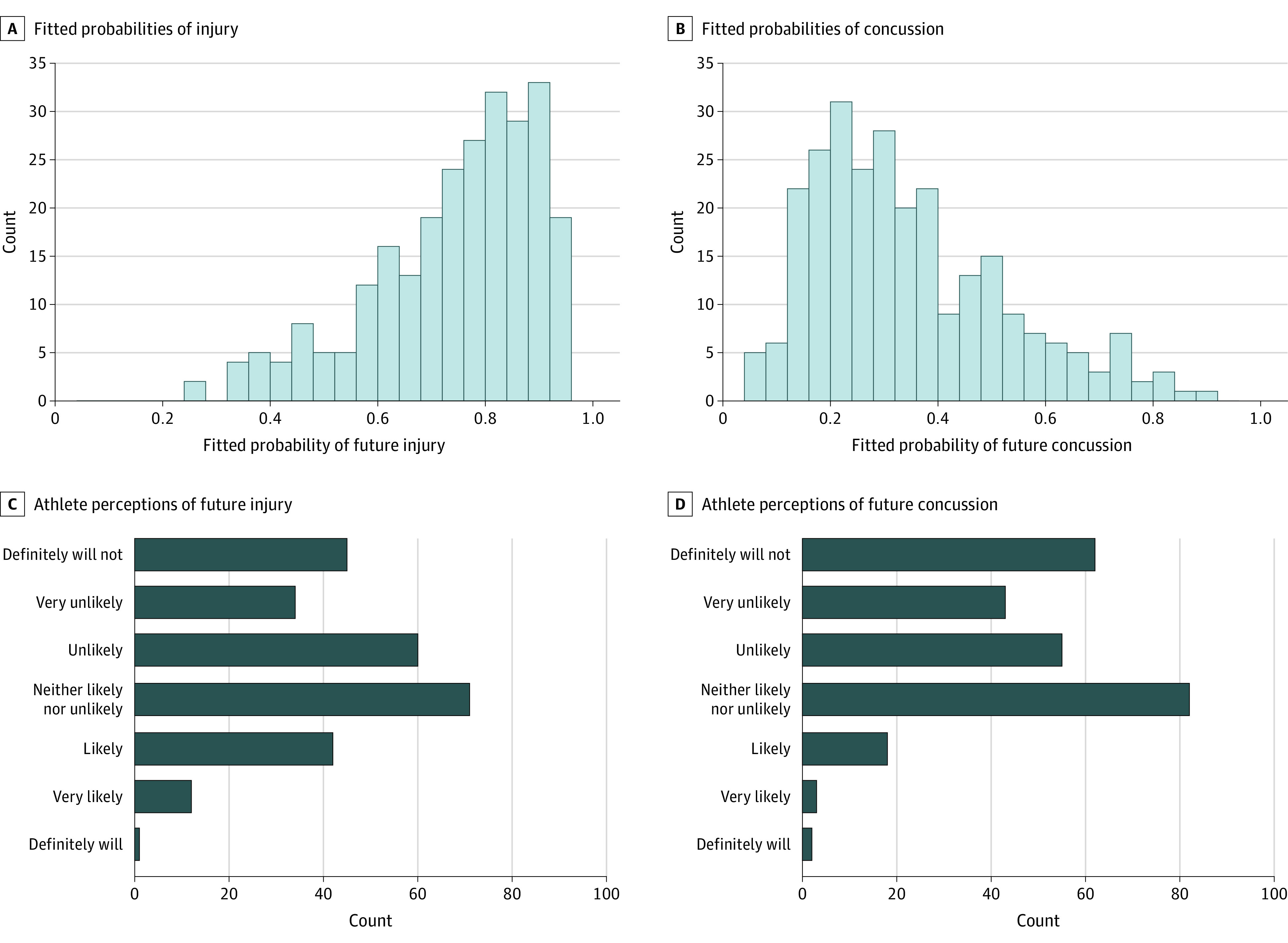
Comparative Distributions of Athlete Perceptions vs Modeled Risks of Future Injury and Concussion

Logistic regression models of single-season injury and concussion had a reasonably good fit (area under the curve for injury, 0.75; area under the curve for concussion, 0.73) (eTable in the Supplement). Fitted probabilities of concussion and injury had different distributions (Figure 2A and B), whereas athletes’ perceptions of injury and concussion risk were distributed similarly (Figure 2C and 2D). In general, the athletes’ perceptions of injury risks appeared skewed toward lower risks than the modeled probabilities.

### Comparing Categorical Modeled and Perceived Risk

**Figure 3.**
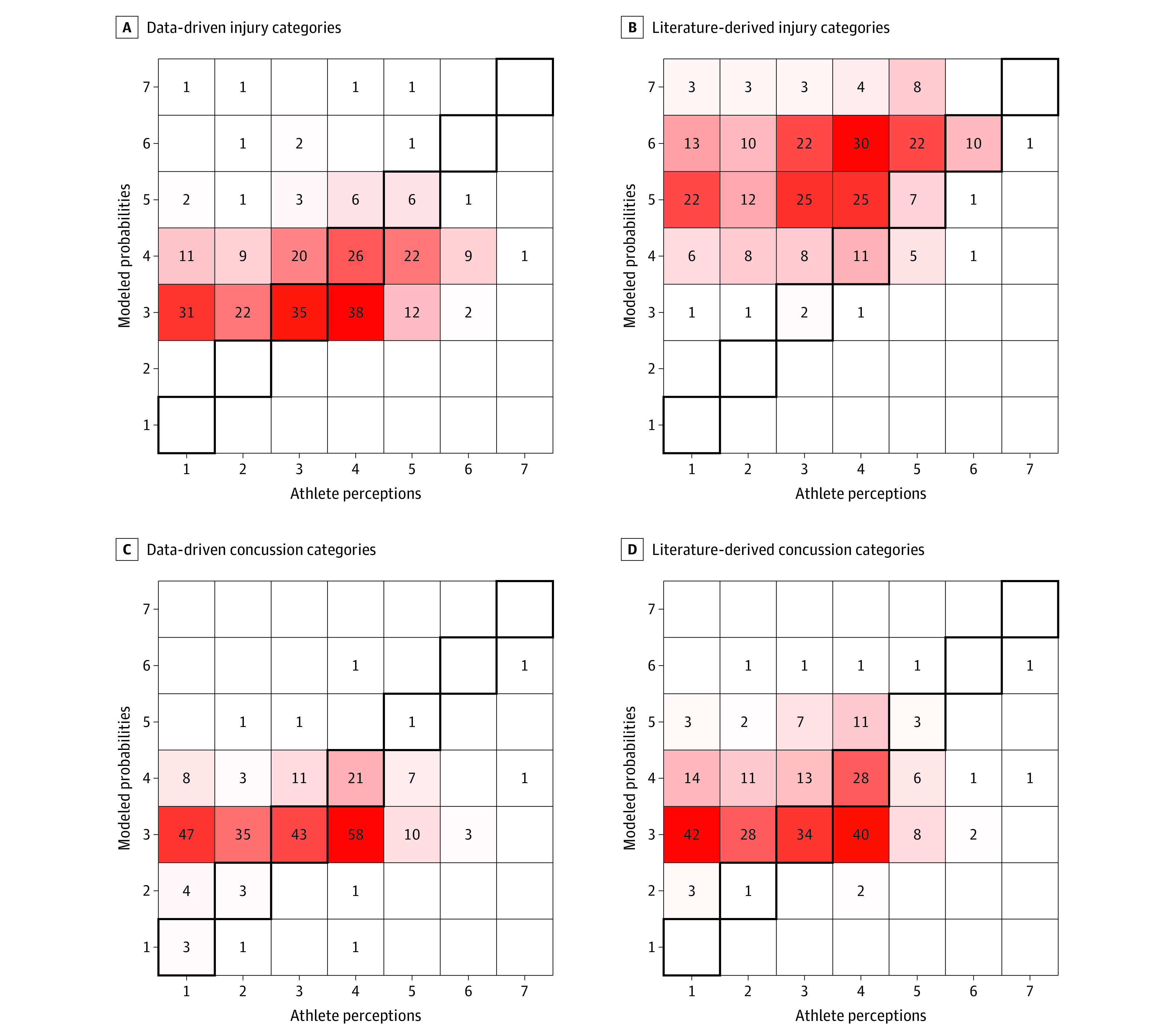
Categorical Comparison of Athletes’ Perceptions vs Modeled Probabilities of Risk of Injury and Concussion The number of athletes in each cell is defined by a combination of athlete perception and modeled probability transformed to categories. Concordant values are in the bolded diagonal cells. Cells above and left of the diagonal represent underestimation of risk compared with the model. Darker red shading indicates more athletes in the cell.

Athletes underestimated their risks of injury and concussion compared with the (categorized) modeled probabilities (Figure 3). Using the literature-derived cut points, 138 athletes (52%) underestimated their risk of concussion (χ^2^ = 43.8; *P* = .04) (Figure 3D) and 226 (85%) underestimated their risk of injury (χ^2^ = 34.2; *P* = .08) (Figure 3B), but the latter difference was not statistically significant. The results were qualitatively similar when we used data-driven cut points; 111 athletes (42%) underestimated their risk of concussion (χ^2^ = 98.6; *P* = .003) (Figure 3C), and 113 (43%) underestimated their risk of injury (χ^2^ = 34.0; *P* = .09) (Figure 3A); the latter difference was not statistically significant.

### Comparing Numerical Modeled and Perceived Risk

Using literature-derived cut points, athletes’ perceptions (transformed to numerical probabilities) were lower than modeled probabilities for injury (91% underestimated; Wilcoxon statistic [W], 7865; *P* < .001) and concussion (63% underestimated; W, 26 768; *P* < .001). Using data-driven cut points, the results were still significant for injury (51% underestimated; W, 28 763; *P* < .001) but not for concussion (60% underestimated; W, 33 788; *P* = .45).

### Characteristics of Athletes Who Misestimated Risk

Exposure to football was associated with misestimation of the risk of injury and concussion. For both injury and concussion, some non–first-year athletes significantly underestimated their risks compared with first-year athletes (Table 2). For concussion only, each additional year that an athlete had participated in contact football was associated with a small overestimation of the risk of concussion (difference, 0.01; *P* = .03). Measured characteristics accounted for a modest proportion of the variation in the gap between modeled and perceived risks (injury *R*^2^, 0.19; concussion *R*^2^, 0.29).

**Table 2.** Characteristics Associated With the Difference Between Athlete Perceptions and Modeled Probabilities of Injury and Concussion Risk^a^

Variable	Injury estimation		Concussion estimation	
	Difference	*P* value	Difference	*P* value
Years of contact football	−0.003	.64	0.01	.03
Year of participation in football				
First	1 [Reference]	1 [Reference]	1 [Reference]	1 [Reference]
Second	−0.04	.49	−0.19	<.001
Third	−0.04	.44	0.05	.28
Fourth	−0.14	.06	−0.03	.63
Fifth	−0.16	.02	−0.03	.60
Mother’s educational level				
High school	1 [Reference]	1 [Reference]	1 [Reference]	1 [Reference]
Some college	0.04	.54	0.01	.79
College degree or higher	−0.004	.95	−0.03	.59
Father’s educational level				
High school	1 [Reference]	1 [Reference]	1 [Reference]	1 [Reference]
Some college	−0.30	.61	0.003	.96
College degree or higher	0.02	.71	0.03	.49
Race/ethnicity				
Other than non-Hispanic White	1 [Reference]	1 [Reference]	1 [Reference]	1 [Reference]
Non-Hispanic White	−0.008	.84	−0.03	.44

^a^ Results are from linear models of perceived minus modeled risk of injury and concussion; difference coefficients represent the difference in modeled minus perceived risk associated with the variable. Literature-derived cut points were used to convert athlete perceptions to probabilities for computation of this difference. Negative coefficients indicate greater underestimation of risk by athletes compared with the modeled probabilities; positive coefficients indicate overestimation.

## Discussion

This survey study suggests that many college football players underestimate the risk of football-related injury generally and concussion specifically. Across analytic strategies, we found that many athletes underestimate risk. The primary analytic strategy suggested that 91% of athletes underestimated their risk of injury and 63% underestimated their risk of concussion. Using a more conservative measurement approach, 43% of athletes underestimated their risk of injury and 42% underestimated their risk of concussion. These findings are in line with previous research suggesting that males, athletes, and individuals participating in the activity they are evaluating tend to underestimate their personal health risks^7^ and raise the possibility that if athletes accurately appraised the risks of football, their decisions around injury reporting and continued participation might change. Although underestimation of personal health risks is common in young adult men, studying this phenomenon in the context of football—a sport in which the risks are presently a topic of societal debate—is, to our knowledge, a novel contribution.

Rates of suspected concussion and nonconcussion injuries were higher than previously published rates of diagnosed injuries in college football.^16^ For comparison, a recent large epidemiologic study found that 5 or 6 concussions are diagnosed per team per season in college football.^17^ In this study, approximately one-third of athletes (34%) indicated that they had sustained at least 1 suspected concussion during the previous football season, or a mean (SD) of 43 (6.5) concussions per participating team. One possible contributing factor is that the sample was drawn from the most competitive division of college football, in which underlying concussion rates may be higher and/or more robust medical oversight may lead to a higher proportion of concussions being diagnosed. Concussion underreporting, which prior research has found to be endemic, may further explain some of the gap between previous estimations of diagnosed concussions and present estimations based on athlete experience.^18,19,20,21,22,23,24,25^

Whether to study athlete-suspected concussions or diagnosed concussions depends on the research question.^26,27^ We sought to understand how athletes’ risk perceptions aligned with their modeled risks and chose to study suspected concussions for 3 main reasons. First, concussion diagnosis depends on volitional symptom disclosure by athletes; thus, estimating the number of future diagnosed concussions depends both on the athletes’ own estimated concussion risk and on internal and external processes leading to diagnosis. Second, athletes’ suspected concussions may more accurately reflect their concussion burden than diagnosed concussions owing to consistent underreporting. Third, comparing past and future risks of suspected concussion is congruent because both rely on a within-athlete understanding of what constitutes concussion. The downsides to the approach used in this study are that we did not know whether athletes’ internal definitions of concussions varied or were medically accurate or what percentage of the athlete-suspected concussions would be diagnosed if reported.

We believe that use of self-reported injury history and demographic and athlete features to model single-season injury risk is appropriate. Previous research has identified factors associated with football athlete injury and injury reporting behaviors. Prior injury and years of participation were found to be factors associated with injury and reporting behaviors among high school football athletes.^28^ Playing position, years on the team, previous injury, and concussion history have been found to be associated with concussion and concussion underreporting in college football athletes.^18,19,20,29^Although retrospective reports of injury are imperfect at the gross level of being able to recall any injury, they may be reliable when considering the previous year.^30^

That athletes underestimated their risk of concussion and injury in this study raises important ethical considerations. What is the threshold for college athletes to be sufficiently informed of the risks and benefits of football to make decisions that align with their values and preferences?^2^ Given the tendency to underestimate personal health risks more broadly, athletes’ underestimation of football-related risk may be no different from the underestimation of other health risks. Alternatively, the risk is higher (more severe or debilitating) than some other health risks, and thus underestimating these risks may undermine the basis of informed consent to participate. In addition, motivated reasoning (eg, wanting an outcome to be true or likely) may be influential in this context. For example, college football athletes may overweight the possibility of a professional football contract when evaluating the risks that they face through collegiate football participation.

Aligning athletes’ risk perceptions with the true underlying risk (as well as possible benefits) is important. One approach is to use knowledge translation efforts to alter risk perceptions. Best practices in health-risk communication should be incorporated into efforts to communicate to athletes the risk of injury, including considerations of numeracy, visual representation, cumulative risk, and communicating small probabilities.^31^ Another approach would be to adopt policies or practices to reduce the true underlying risk such that it aligns with athlete expectations. Examples include reducing or altering high-risk plays within football (eg, kick off), reducing the amount or intensity of contact sustained by athletes, or ensuring adequate access to independent athletic medicine clinicians.^32,33^ Regardless of the approach, efforts should be made to ensure that athletes have an appropriate understanding of the risks that they incur through sport to empower their informed decision-making. Future research should specifically evaluate such efforts in the college athletics environment.

The findings of this study take on a new context as decisions are being made about the return of college football amid the COVID-19 pandemic. College football players have returned to college campuses to participate in football activities during a time in which an infectious disease pandemic is surging in areas of the US. Without significant intervention, participation in football is incongruous with many infection-mitigation strategies—it involves large groups and physical contact without protective masks, in some cases with fans in the stands. Given this study’s findings that college football players underestimated their personal risk of relatively well-understood risks of concussion and other injury, it seems unlikely that they accurately estimate their personal risk of a less well-understood infectious disease. Exposing these emerging-adult athletes to the risks of COVID-19 without additional countervailing benefit to the athlete, knowing that they may underestimate the health risks the disease poses to them, is concerning and possibly unethical.

### Limitations

This study has limitations. It focused on emerging-adult football players in the competitive college context and cannot be generalized to risk perceptions of athletes of other ages or stakeholders of other sports (eg, parents of youth athletes). Although participating and nonparticipating teams were balanced on nearly all observable characteristics measured, unmeasured factors may have differed (eg, team-level differences in injury or concussion rates), including factors that may have been associated with outcomes of interest (eg, underlying rates of learning disabilities affecting risk of concussion). Estimating athletes’ future injury risk based on their injury history assumes reliable injury history information, reasonable predictors of risk, persistence of the impact of risk factors, and reasonable model fit. Injury rates may be associated with factors not included in this study’s models (eg, training regimens, coaching staff). On the basis of previous research, we would expect underreporting of injury, and risk of injury may vary in ways we could not observe. Converting between modeled probabilities of injury and athlete Likert scale predictions requires assumptions about what respondents meant when they selected Likert categories. We used 1 study^14^ in the broader literature^34,35^ to guide the literature-derived cut points. Our use of both literature-derived and data-driven cut points indicated substantially similar conclusions; that is, even using a conservative approach to assessment, many athletes underestimated their personal risks. In addition, we did not investigate whether athletes’ decisions would change if their perceived risks were more aligned with calculated risks.

## Conclusions

This study suggests that athletes underestimate the risks associated with participation in football. Knowledge translation efforts appear to be needed to help athletes more accurately appraise their risk of injury and inform risk-related decision-making, including injury reporting. Future work evaluating the mechanisms underlying this underestimation and the best ways to align risk perceptions with true underlying risks is warranted. However, it is necessary to consider what it means for athletes to be sufficiently informed in the context of college football and the level of risk that is acceptable for participation in a sport. This is made more salient with athletes assuming less well-known risks related to COVID-19 as they return to participation in college football amid the pandemic.

## Notes

Supplement. **eAppendix.** Details of Transformation Between Numerical and Categorical Risks **eTable.** Characteristics Associated With Single-Season Risk of Injury and Concussion **eReference**
