# Supplementary material for: Accuracy of US College Football Players’ Estimates of Their Risk of Concussion or Injury
Source: JAMA Netw Open. 2020 Dec 29;3(12):e2031509. doi: 10.1001/jamanetworkopen.2020.31509 (PMC7772718; doi:10.1001/jamanetworkopen.2020.31509)
Supplement: Supplement. — eAppendix. Details of Transformation Between Numerical and Categorical Risks eTable. Characteristics Associated With Single-Season Risk of Injury and Concussion eReference [file jamanetwopen-e2031509-s001.pdf]

Supplementary Online Content

Baugh CM, Kroshus E, Meehan WP III, McGuire TG, Hatfield LA. Accuracy of college football players' estimates of their risk of concussion or injury. *JAMA Netw Open*. 2020;3(12):e2031509. doi:10.1001/jamanetworkopen.2020.31509

eAppendix. Details of Transformation Between Numerical and Categorical Risks

eTable. Characteristics Associated With Single-Season Risk of Injury and Concussion

eReference

This supplementary material has been provided by the authors to give readers additional information about their work.

© 2020 Baugh et al. *JAMA Network Open*.

eAppendix. Details of transformation between numerical and categorical risks

We used two different methods to transform between modelled probabilities (continuous values between 0 and 1) and athlete perceptions (measured on a 7-point Likert scale).

Literature-Derived
First, we used existing literature[1] on people's qualitative interpretations of probabilities to transform athlete perceptions into numerical probabilities: Definitely won't=0, Very Unlikely=0.05, Unlikely=0.20, Middle Category=0.50, Likely=0.70, Very Likely=0.90, Definitely Will=1. Going in the other direction, we transformed modeled probabilities into seven ordinal categories using the following cut points: (0, 0.025, 0.075, 0.35, 0.6, 0.8, 0.95, 1).

Data-Driven
Second, we used a transformation that minimized the differences between the modeled values and athletes' perceptions. We searched over the space of cut points to find one that minimized the sum of the absolute distance between the categorized probabilities and athlete perceptions. For a set of cut points, each modeled probability can be transformed into one of 7 categories, represented as an integer: Definitely won't=1, Very Unlikely=2, Unlikely=3, Middle Category=4, Likely=5, Very Likely=6, Definitely Will=7. We computed the absolute distance between the integer representation of the athlete's actual Likert response and the integer category of the transformed modelled probability. Summing these absolute differences over athletes, we get a score for how closely the categorized probabilities match the athlete ratings. Minimizing this score produced data-driven cut-points for injury (0.09, 0.24, 0.81, 0.93, 0.98, 0.99) and concussion (0.06, 0.11, 0.49, 0.8, 0.87, 0.93). In addition, we transformed athlete perceptions into numerical values using the midpoints of each category.

© 2020 Baugh et al. *JAMA Network Open*.

eTable. Characteristics associated with single-season risk of injury and concussion

	Injury Model		Concussion Model	
Variable	*Log OR*	*p-value*	*Log OR*	*p-value*
Years of Contact Football	0.08	0.15	0.02	0.71
Position—OL	REF	REF	REF	REF
Position—DL	0.49	0.40	0.20	0.71
Position—DB	1.04	0.07	0.13	0.79
Position—LB	0.08	0.15	-0.10	0.85
Position—RB	1.30	0.10	-0.06	0.92
Position—QB	-0.19	0.80	-1.07	0.18
Position—TE	1.06	0.24	-0.74	0.32
Position—WR	0.82	0.14	-0.69	0.19
Position—ST	-0.29	0.67	-1.46	0.10
First-Year Athlete	REF	REF	REF	REF
Second-Year Athlete	0.10	0.81	0.89	0.03
Third-Year Athlete	0.22	0.33	-0.38	0.41
Fourth-Year Athlete	0.02	0.97	-0.04	0.95
Fifth-Year Athlete	0.42	0.56	-0.70	0.25
Team 1	REF	REF	REF	REF
Team 2	-0.12	0.84	0.54	0.21
Team 3	-0.95	0.04	0.09	0.82
Team 4	-0.72	0.12	-0.32	0.43
Role—First Team	REF	REF	REF	REF
Role—Second Team	0.17	0.69	0.61	0.11
Role—Third Team	-0.15	0.84	0.11	0.85
Role—Practice Squad	-0.38	0.41	-0.66	0.24
Role—Redshirted	-0.53	0.12	-0.18	0.71
Previous Injury History	0.11	0.02	0.06	0.03
Table Caption. Coefficients are log odds ratios from logistic regression models containing all predictors in the table. Positive coefficients indicate higher probability of injury or concussion. AUC for the injury model = 0.75. AUC for the concussion model = 0.73.				

© 2020 Baugh et al. *JAMA Network Open*.

eReference

1. Benthin A, Slovic P, Severson H. A psychometric study of adolescent risk perception. *J Adolesc*. 1993;16(2):153-168. doi:10.1006/jado.1993.1014

© 2020 Baugh et al. *JAMA Network Open*.
